# Development of Photo-Active Chitosan-Based Films with Riboflavin for Enhanced Antimicrobial Food Packaging Applications

**DOI:** 10.3390/molecules30214166

**Published:** 2025-10-23

**Authors:** Jessica Genovese, Daniele Maria Martins, Tiziana Silvetti, Milena Brasca, Daniela Fracassetti, Gigliola Borgonovo, Stefania Mazzini, Sara Limbo

**Affiliations:** 1 Department of Food, Environmental and Nutritional Sciences (DeFENS), Università degli Studi di Milano, Via G. Celoria 2, 20133 Milan, Italy; jessicagenovese1988@gmail.com (J.G.); daniele.martins@unimi.it (D.M.M.); daniela.fracassetti@unimi.it (D.F.); gigliola.borgonovo@unimi.it (G.B.); stefania.mazzini@unimi.it (S.M.); 2 Institute of Sciences of Food Production, National Research Council (CNR-ISPA), Via G. Celoria 2, 20133 Milan, Italy; Tiziana.Silvetti@cnr.it (T.S.); milena.brasca@cnr.it (M.B.)

**Keywords:** chitosan-based films, riboflavin photosensitizer, photodynamic inactivation, antimicrobial packaging, food shelf life

## Abstract

This study reports the development of chitosan-based (CS) films incorporating riboflavin (RF) as a natural photosensitizer to create sustainable, light-activated antimicrobial packaging materials. The films were prepared by solvent casting, and their photochemical behavior under blue LED light (450 nm) was investigated, including RF photodegradation kinetics and structural changes in the film-forming solution analyzed by ^1^H NMR spectroscopy. Mechanical, thermal, optical, and barrier properties were also characterized to assess packaging suitability. Upon illumination, CS/RF films generated reactive oxygen species, particularly singlet oxygen (^1^O_2_), leading to visible color changes and significant antimicrobial activity against *Pseudomonas fluorescens*. Bacterial growth was reduced by up to 97% after 120 min of irradiation (0.92 J cm^−2^), with efficacy observed at both room temperature and 4 °C. The incorporation of RF did not alter the films’ mechanical properties, while thermal stability was preserved, optical transparency was modulated, and excellent oxygen barrier performance was maintained, although water vapor permeability remained moderate. These findings demonstrate that CS/RF films combine functionality and sustainability, offering a promising strategy for extending food shelf life through light-activated antimicrobial action. Validation under real storage conditions is recommended to confirm their potential in diverse food systems.

## 1. Introduction

Photodynamic inactivation (PDI) of microorganisms represents an innovative and environmentally friendly approach for enhancing food preservation using natural photosensitizers. PDI employs visible light at specific wavelengths to activate these photosensitizers, which generate reactive oxygen species (ROS) capable of inducing oxidative damage in microbial cells, thus leading to their inactivation [1,2]. This technology allows for effective control of foodborne pathogens including Gram-positive bacteria like *Staphylococcus aureus* and Gram-negative bacteria such as *Escherichia coli* [3,4,5] while maintaining food quality, making it increasingly relevant in food safety and preservation [6]. This is particularly relevant in the context of the growing consumer demand for clean-label products that avoid synthetic additives, encouraging the use of natural light-sensitive molecules, which can replace synthetic alternatives in various applications [7].

Driven by this trend, the incorporation of edible photosensitizers into bio-based polymers for packaging applications has emerged as a promising strategy to improve food safety and extend shelf life. These materials can respond to external environmental stimuli (e.g., light, oxygen, moisture), enabling dynamic interactions with the food environment to preserve quality and safety throughout shelf life [8,9,10]. Such stimuli-responsive systems enable the development of advanced packaging solutions with functionalities activated by environmental changes. Among them, photo-active packaging systems are particularly promising for applications involving perishable foods, such as fruits, vegetables, meats, and dairy products [11].

Edible photosensitizers, including chlorophyllin, riboflavin, and curcumin, can thus impart photoactivated antimicrobial functionality to biopolymeric films, with riboflavin being one of the most extensively studied molecules in this context [12,13,14,15,16,17,18]. Notably, riboflavin (RF), a water-soluble vitamin, has demonstrated efficacy in antimicrobial photodynamic therapy [19] and it can inactivate a broad spectrum of microorganisms, including both Gram-positive and Gram-negative bacteria [20]. When excited by light, RF generates reactive intermediates, such as ROS which include singlet oxygen, hydrogen peroxide, hydroxyl radicals, and superoxide anion, able to attack cellular DNA, RNA, proteins and lipids and result in the destruction of cells [21,22]. In fact, riboflavin (RF) is well-documented to undergo photoactivation through multiple mechanisms involving both its excited singlet and triplet states [23,24,25,26].

Upon exposure to light, RF is rapidly excited from the ground state (S_0_) to its singlet state (S_1_), with a lifetime of approximately 5 ns, followed by transition to the triplet state (T_1_), which has longer lifetime of around 10 µs. These excited states facilitate different photochemical reactions. Specifically, the excited triplet state of RF is responsible for generating singlet oxygen (^1^O_2_) through the triplet oxygen annihilation mechanism, a key process in photosensitized reactions. Riboflavin in the T_1_ state also leads to the formation of major photoproducts, such as formylmethylflavin (FMF), lumichrome (LC), and lumiflavin (LF), the latter being preferentially derived at alkaline pH [26]. On the other hand, the excited S_1_ state is involved in the formation of LC and cyclodehydroriboflavin (CDRF) [27]. The rapid excitation and subsequent energy transitions allow RF to participate in these reactions within a very short timeframe. These photochemical reactions are influenced by several factors, with the emission characteristics of the radiation (i.e., irradiance energy and wavelength) being particularly important. Su et al. (2021) successfully developed an active film by incorporating a high concentration of RF (3% *w*/*w*) into a chitosan film-forming solution, demonstrating enhanced antimicrobial effect in vitro and on fresh salmon, using high-radiation doses (3.42–13.68 J/cm^2^) of a blue LED light [15].

Although the effects of LED light on food preservation have been extensively studied, the specific impact of LED light doses that are more realistically comparable to those encountered during food storage on ROS generation and microbial inactivation remain underexplored. This knowledge gap highlights the need for focused investigation to better understand the interplay between light-induced ROS and microbial control, which could have significant implications for enhancing food safety and extending shelf life. The incorporation of photosensitizers into biopolymers presents some challenges, as their efficiency can be influenced by solubility limitations and the surrounding conditions within the polymer matrix. This underscores the importance of carefully selecting the biopolymer and the photosensitizer optimizing loading and formulation parameters to maximize the antimicrobial activity of photosensitizers in food packaging applications. To address these challenges, chitosan emerges as a promising option [28]. The presence of amino groups in chitosan imparts distinct functional properties, including solubility, film-forming ability, viscosity modulation and ion-binding capacity [29]. Moreover, recent studies [30,31] show that higher molecular weight (MW) enhances tensile strength and stiffness of chitosan film through stronger chain entanglement and hydrogen bonding, whereas lower MW yields more flexible but weaker films. The MW distribution, degree of deacetylation, and crystallinity further influence these properties by modulating hydrogen bonding and chain organization. Plasticizers such as glycerol disrupt intermolecular bonds, increasing flexibility but reducing stiffness, thereby highlighting their crucial role in film formulation. Furthermore, the cationic amino groups along the chitosan backbone contribute to its antimicrobial activity, which has been demonstrated against bacteria, yeast, molds, and fungi [32,33]. Overall, the tunable structure–property relationships of chitosan underline its versatility for both active and edible packaging applications [34,35].

Building on this foundation, the present study developed a photo-active food packaging material by incorporating RF into a chitosan/glycerol (CS) film-forming solution. The resulting CS/RF films are designed to exhibit photo-active antimicrobial properties, offering a sustainable and functional approach to food preservation. The study systematically investigates the photochemical behavior of RF in both CS film-forming solutions and solid films, emphasizing its photodegradation kinetics and ROS generation under blue LED irradiation at 450 nm, corresponding to the RF absorption peak. Additionally, the physical, chemical, and barrier properties of the CS/RF films were characterized to assess their suitability as packaging materials. Finally, the antimicrobial efficacy of the films was assessed in vitro, to evidence if RF-mediated photodynamic inactivation can effectively suppress *Pseudomonas fluorescens* microbial growth and position these films as biodegradable alternatives to petroleum-based plastics.

## 2. Results and Discussion

### 2.1. Riboflavin Behavior and Kinetics Under Light Exposure

The photodynamic treatment of film-forming solutions and films was performed using a customized multispectral LED system with wireless control, allowing precise adjustment of light intensity, wavelength, and exposure time (for more details, see Section 3.3). The UV–Vis spectrophotometric response of riboflavin (RF) in the 300–600 nm range was monitored during light exposure at room temperature in both the CS/RF system (i.e., the film-forming solution containing chitosan, glycerol and riboflavin, in acetic acid at 1% *v*/*v*) and the RF system (the same solution without chitosan).

**Figure 1:**
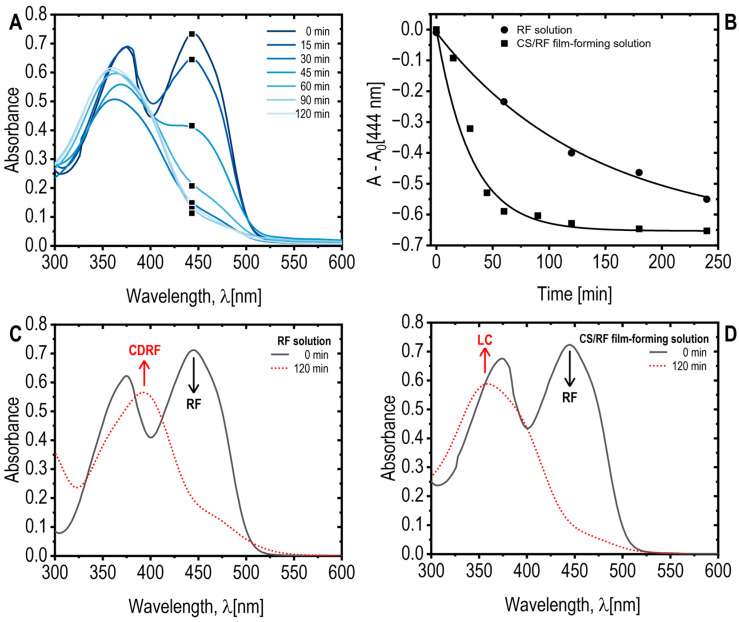
(**A**) Absorption spectra of the CS/RF film-forming solution exposed to blue LED light for various durations. Note that only a selected wavelength range and specific exposure times are displayed for clarity; (**B**) Absorbance of RF acetic acid solution and CS/RF film forming solution at 444 nm, subtracting the absorbance at 0 min (A_0_), as a function of blue LED light exposure time. Selected data points are marked with black square on the corresponding curves in the A plot; (**C**) Absorption spectra of RF acetic acid solution, and (**D**) absorption spectra of CS/RF film-forming solution, both unexposed (0 min) and exposed to blue LED light for 120 min. The riboflavin (RF) and its photoproducts–cyclodehydroriboflavin (CDRF) and lumichrome (LC)–are indicated.

Figure 1A presents the spectral changes in the CS/RF solution, where RF absorption bands at 370 nm and 444 nm were observed [36,37]. Upon blue LED exposure, the absorbance at 444 nm decreased by 50% within 40 min, reaching a maximum reduction at 60 min of light exposure (0.46 J cm^−2^ of energy dose) (Figure 1B). Prolonged irradiation shifted the UV absorption at 370 nm to 354 nm, with an isosbestic point at 402 nm (Figure 1A), suggesting structural modification of RF while maintaining reaction stoichiometry [38]. In the absence of chitosan, RF degradation led to the formation of a new band at 410 nm (Figure 1C), indicative of a photoproduct identified as CDRF [27,36]. Conversely, in the CS/RF solution, a new band at 356 nm (Figure 1D) signified the formation of lumichrome (LC), as previously reported [36,39]. These findings highlight the influence of chitosan on RF photodegradation pathways and photoproduct formation. In the CS/RF film-forming solution, our results indicate that RF predominantly undergoes photodegradation to form LC, suggesting that the photoreduction pathway is favored over the photoaddition pathway (i.e., that forms CDRF) [27].

To investigate the structural modifications of riboflavin (RF) within the film-forming solution, the CS/RF system was analyzed using ^1^H NMR spectroscopy. All samples were prepared under the same conditions used for film preparation (Section 3.4.2). Control samples of RF and CS/RF film-forming solutions, maintained in the dark (0 min), showed no differences in RF signals, confirming that all observed reactions were induced by light exposure).

**Figure 2:**
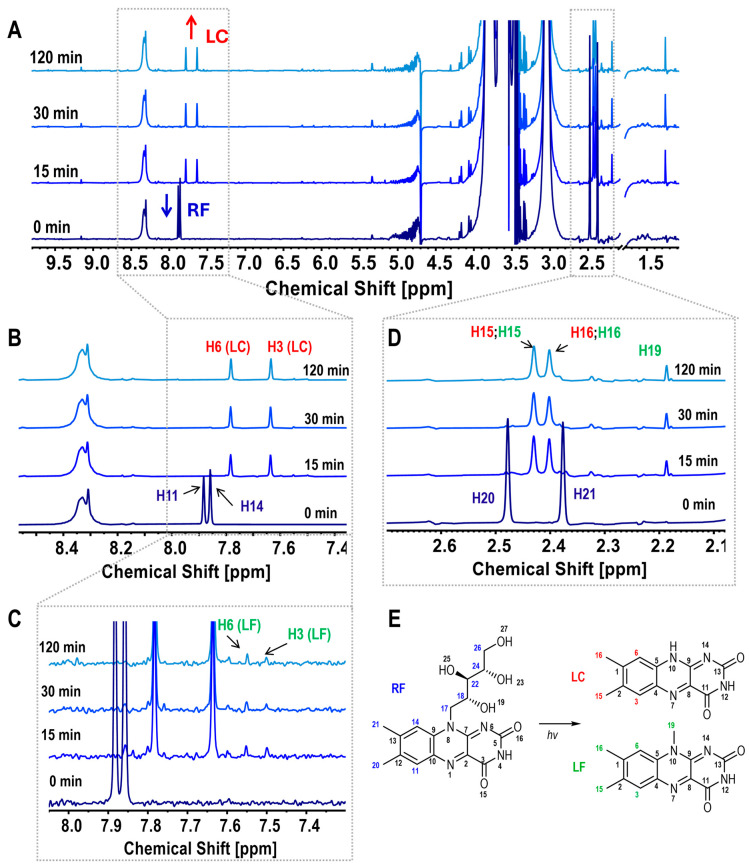
(**A**) ^1^H NMR spectra of CS/RF film-forming solutions at various irradiation times, with labeled signals for RF and LC; (**B**) Expanded spectra highlighting RF peaks (H11, H14) and LC peaks (H6, H3); (**C**) Expanded spectra highlighting LF peaks (H6, H3); (**D**) Expanded view of assigned methyl peaks for RF (H20, H21), LC (H15, H16), and LF (H15, H16, H19); (**E**) Schematic representation of riboflavin (RF) photodegradation, illustrating the formation of lumichrome (LC) and lumiflavin (LF) with corresponding assignment numbers.

The ^1^H NMR spectra of the CS/RF film-forming solution at various irradiation times (0, 15, 30, and 120 min) are presented in Figure 2, with molecular assignments for RF degradation products shown in Figure 2E. The assignments are based on the study by Bliumkin et al. (2016), which identified lumichrome (LC) and lumiflavin (LF) as the primary photoreduction products of RF, along with minor photodegradation byproducts [40]. Upon irradiation, the RF signals in the CS/RF solution exhibited a decrease in intensity, particularly in the aromatic peaks at δ 7.86 and 7.88 ppm (H11 and H14, Figure 2A,B), and the methyl singlet peaks at δ 2.38 and 2.48 ppm (H20 and H21, Figure 2A,D). Simultaneously, new singlet peaks characteristic of LC emerged at δ 7.78 and 7.64 ppm (H6 and H3, in Figure 2A,B), along with overlapping LC and LF signals at δ 2.43 and 2.40 ppm (H15, H16, Figure 2A,D). Additional peaks at δ 7.55 and 7.50 ppm (expanded in Figure 2C) and a methyl signal at δ 2.19 ppm (H19) were attributed to LF (Figure 2C). The RF signals, specifically H11 and H14 (Figure 2A,B), exhibited rapid degradation, with the most significant changes occurring within the first 15 min of irradiation. Beyond 30 min, no substantial differences were observed, as reflected in the degradation curves of H11 and the formation of H3 from LC (Figure S1).

The interaction between RF and the chitosan (CS) chain was also examined. The ^1^NMR spectra of CS and CS/RF film-forming solutions exhibited characteristic signals of N-acetylglucosamine (GlcNAc) and glucosamine (GlcN) units of chitosan [41]. No significant changes were observed in CS peaks when comparing CS and CS/RF solutions (Figure S2), indicating that RF photodegradation did not alter the co-unit distribution within the CS macromolecular structure.

### 2.2. CS/RF Film as Potential Food Packaging Material

**Figure 3:**
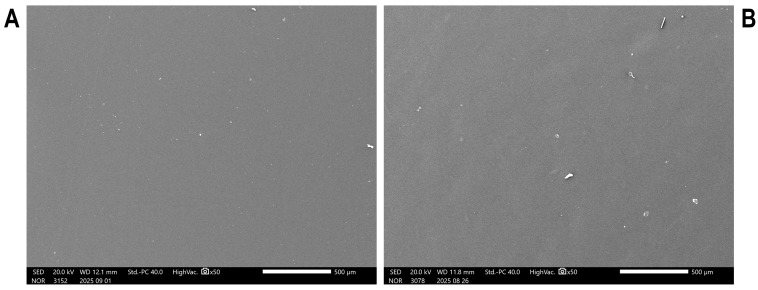
Scanning electron microscopy (SEM) micrographs of (**A**) CS and (**B**) CS/RF films.

The SEM micrographs of the CS and CS/RF films are presented in Figure 3. These images provide insight into the surface morphology of the chitosan-based films and the effect of RF incorporation on the polymer matrix structure. The CS film (Figure 3A) exhibits a homogeneous and smooth surface with no evident porosity. This morphology is characteristic of chitosan, which typically forms compact and continuous films due to strong intermolecular interactions and a uniform polymer chain arrangement. Upon the addition of RF (Figure 3B), the surface remains uniform, with no apparent aggregates or discontinuities. The similar morphological features observed in the CS/RF film suggest good dispersion of RF within the polymer network, indicating compatibility between the components.

**Figure 4:**
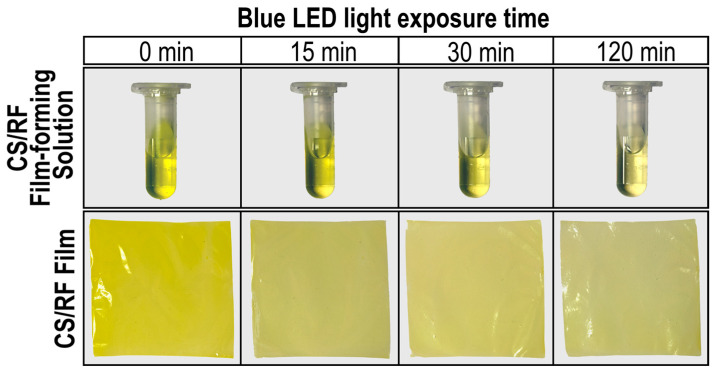
Photographs of CS/RF film-forming solutions and CS/RF films exposed to blue LED light for different durations.

Figure 4 shows photographs of the CS/RF film-forming solution and films subjected to photodynamic treatment using blue LED light. With increasing exposure time, a noticeable fading in color was observed in both the solution and the film. These changes were quantitatively analyzed by measuring the CIE color parameters L*, a*, and b* to objectively track the extent of color alteration of the film during light exposure. In this color space, L* represents lightness, a denotes the position between green (negative values) and red (positive values), and b* represents the position between blue (negative values) and yellow (positive values). This standardized system allows for an accurate and reproducible quantification of color variations observed visually. The results, summarized in Table 1, showed clear trends in the color parameters of the CS/RF film as a function of LED light exposure. Specifically, the a* values increased, indicating a reduction in the ‘green’ component, while the b* values decreased, reflecting a reduction in the ‘yellow’ component of the film color. This combination of changes in the a* and b* values reflect a gradual fading of the initial yellow-green hue of the films, corroborating the visual observations of color fading shown in Figure 4. Furthermore, the total color change (ΔE) and yellowness index (YI), calculated as described in Section 3.6.1, supported this trend—higher ΔE and lower YI values were associated with longer exposure times, underscoring the progressive shift in color as a function of accumulated light energy.

In addition to color changes, the optical transparency of both the CS film and the CS/RF film was evaluated following exposure to various durations of blue LED light. Optical transparency is a critical attribute for consumer acceptance, as it allows the visual inspection of food freshness and appearance prior to purchase [42]. The most used parameter for evaluating the optical transparency of food packaging materials is transmittance across the UV–Vis range (200–800 nm).

**Figure 5:**
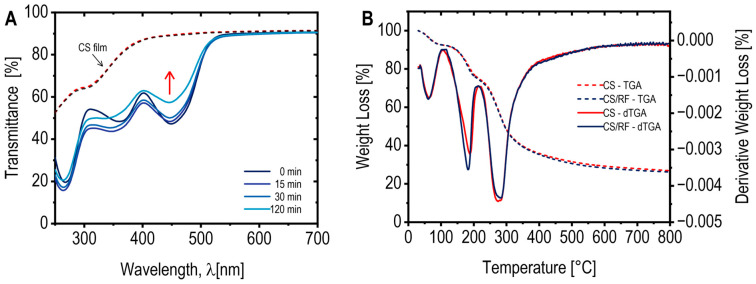
(**A**) UV/Vis transmittance spectra of the CS film (dotted lines) before and after 120 min of blue LED light exposure (note both lines are overlapping), and CS/RF films (solid lines) exposed to blue LED light for various durations; (**B**) TGA curves (dotted lines) and derivative thermogravimetric profiles (dTGA) (solid lines) of the CS film and CS/RF film before light exposure.

Our results indicate that incorporating RF into the CS-based matrix (to produce CS/RF film) reduces light transmission in the UV–Vis region up to 500 nm (Figure 5A). Similar trends have been reported with increasing amounts of RF added to CS polymers [15]. This reduction in light transmission could play a critical role in blocking potentially harmful UV–Vis radiation from reaching packaged food products, particularly during the early stages of shelf-life. However, with prolonged light exposure, a gradual increase in light transmission at 450 nm (correlated with the concurrent degradation of RF) is observed, although the CS/RF film consistently exhibited lower transmittance (in the region below 500 nm) compared to the pure CS film. It is important to note that opacity measurements are dependent on the film’s thickness. In our study, the thickness of both CS and CS/RF films was 0.024 ± 0.004 mm, yielding a calculated opacity value of 1.95 ± 0.028 for wavelengths above 600 nm, indicating minimal light barrier properties in this range. The optical properties of the CS/RF film, characterized by its ability to modulate light transmission over time, may provide an optimal balance between protecting food products from light exposure and maintaining visibility throughout their shelf-life.

From an application perspective, large-scale production of packaging materials requires specific technological properties, followed by the barrier properties and mechanical resistance to extend the food product shelf-life and logistic [42]. One critical technological feature of polymeric films is their thermal stability, the assessment of which also provides insights into whether the incorporation of additives into the polymeric matrix affects the material degradation during production [43].

The thermal stability of the CS and CS/RF films was assessed using thermogravimetric analysis (TGA), and the resulting TGA-dTGA curves are presented in Figure 5B. Both films exhibited an initial weight loss in the temperature range of 30 to 100 °C, which corresponds to the evaporation of adsorbed water and volatile components. The second weight loss occurred between 100 and 250 °C (T_max_ 190 °C) and can be attributed to the evaporation of glycerol incorporated into the film formulations as a plasticizer. The third and major weight loss region, observed from 250 to 400 °C (T_max_ 275 °C), was associated with the thermal degradation of chitosan, involving its decomposition into ammonia, carbon monoxide, and carbon dioxide [43]. The overall thermal behavior of the CS/RF film closely resembled that of the pure CS film, suggesting that the incorporation of RF at a concentration of 60 mg L^−1^ did not significantly influence the thermal stability of the material.

For effective food preservation, the barrier properties of polymeric films are critical, as the packaging must slow the transfer of molecules such as O_2_, CO_2_, water vapor, organic vapors, or liquids between the food and its surrounding environment [44]. Chitosan films are known for their excellent oxygen barrier performance, although their water vapor barrier capabilities are relatively limited due to their hydrophilic nature [45]. In this study, the oxygen and water vapour permeability of CS and CS/RF films were evaluated (measurement curves are provided in Supplementary Materials, Figure S3), and Table 2 reports the oxygen transmission rates (O_2_TR) and water vapor transmission rates (WVTR), along with the calculated permeability coefficients (KPO_2_ and KPWV) for both films.

The results indicate that the CS and CS/RF films exhibit similar values for both transmission rates and permeability coefficients, with no statistically significant differences. The KPO_2_ values obtained for CS and CS/RF were approximately 100 cm^3^ µm m^−2^ day^−1^ bar^−1^, which are substantially lower than those of common synthetic packaging materials such as LDPE (2 × 10^5^ cm^3^ µm m^−2^ day^−1^ bar^−1^, at 23 °C) or amorphous PET (4 × 10^3^ cm^3^ µm m^−2^ day^−1^ bar^−1^, at 23 °C) [46], confirming the excellent oxygen barrier performance of chitosan. The results reinforce the suitability of the CS/RF film for food preservation applications, as it effectively limits oxygen diffusion and, consequently, oxidative reactions that drive quality degradation and microbial growth. However, the moderate KPWV values of the CS and CS/RF films (approximately 1.60 × 10^6^ g µm m^−2^ day^−1^ bar^−1^) reflect the hydrophilic nature of the polymer matrix. These values are substantially higher than those of conventional synthetic barriers such as LDPE which typically exhibit KPWV values below 1.0 × 10^4^ g µm m^−2^ day^−1^ bar^−1^ [46].

As described by Aguirre-Loredo et al. (2016), the water vapor permeability of chitosan films increases with rising RH, a phenomenon attributed to the plasticization of the structural matrix [47]. The amino and hydroxyl groups in the chitosan chain serve as binding sites for water molecules, which, when absorbed, plasticize the amorphous regions of the polymer network, resulting in a more flexible structure and promoting internal rearrangement. It is important to highlight that comparisons of permeability values with those reported in literature should be made cautiously, as the water vapor permeability of chitosan films is influenced by factors such as molecular weight, degree of deacetylation, chitosan content, and the specific conditions under which measurements are conducted [44].

**Figure 6:**
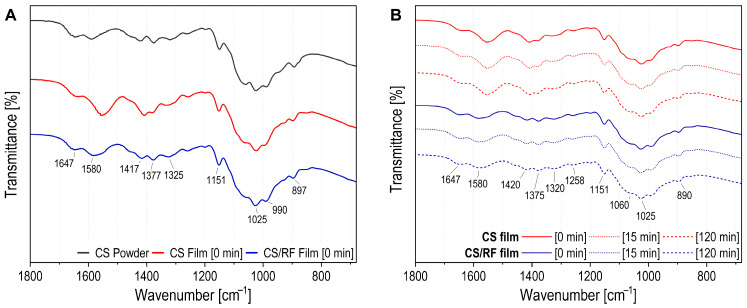
ATR-FTIR spectra and zoomed-in fingerprint region for CS powder, CS and CS/RF films (**A**) before irradiation (0 min); and (**B**) at different irradiation times (0, 15 or 120 min).

ATR-FTIR spectroscopy was employed to investigate the structural changes in the CS/RF film during photodynamic treatment. Spectra were collected before blue LED exposure (Figure 6A and Figure S4a) and after 15 and 120 min of irradiation (Figure 6B and Figure S4b). For comparison, spectra of chitosan powder (10–20 kDa, ≥90% DD) and the CS film were also recorded. Figure 6 presents the fingerprint region (1800–680 cm^−1^), while the full spectra are shown in Figure S4. The band assignments are summarized in Table S1.

In the ATR-FTIR spectrum of CS powder, peaks at 3356 and 3292 cm^−1^ correspond to N-H and O-H stretching, while bands at 2921 and 2872 cm^−1^ are attributed to *ν_s_*(CH) and *ν_as_*(CH) in CH_2_OH of the pyranose ring. These bands are also observed in CS and CS/RF films (Figure S4a). The CS film shows broader peaks in this region, suggesting stronger hydrogen bonding in films without RF [48]. The CS/RF film is dominated by chitosan bands, as the biopolymer is the major component and contains more functional groups than RF, resulting in stronger IR absorption (Figure S4a). In the fingerprint region (Figure 6A), the CS powder spectrum displays a peak at 1646 cm^−1^ for ν(C=O) in amide group (amide I). This band shifts and broadens in the CS/RF film and sharpens at 1635 cm^−1^ in the CS film. The peak at 1589 cm^−1^ corresponds to -NH_2_ bending in 2-aminoglucose, overlapping with N-H bending (amide II) [49]. The CS film exhibits a significant shift in the N-H bending band, observed at 1557 cm^−1^ [50].

The impact of irradiation on the surface of CS and CS/RF films was assessed, and the corresponding spectra obtained after 0, 15 and 120 min of blue LED light exposure are presented in Figure 6B and Figure S4b. Bands in the 3000–3500 cm^−1^ range correspond to OH stretching of free water and pyranose ring OH groups, overlapping with NH stretching of amide A (Figure S4b). Intensity variations observed in the CS and CS/RF films over time may be attributed to changes in water content or water–biopolymer interactions. Bands at 1420 and 1320 cm^−1^ correspond CH_2_ bending in CH_2_OH, ν_s_(CH_3_) in amide III, and CH_2_ wagging with in-plane OH deformation (Figure 6B). The peak at 1375 cm^−1^ represents CH_3_ in-plane scissoring in NHCOCH_3_. The amide III band, attributed to ν(C-N) and N-H in-plane bending is observed at 1258 cm^−1^. The ν(C-O) band at 1151 cm^−1^ for the C-O-C bridge in chitosan shows no displacement. The peak at 1060 cm^−1^ corresponds to ν(C-O) from C3-OH vibration in secondary alcohol, while the peak at 1025 cm^−1^ is linked to C6-OH vibration in primary alcohol (Figure 6B) [51]. The band assignments in Figure 6B and Figure S4b indicate that the peak patterns in both CS and CS/RF films remained consistent across varying irradiation durations. Thus, despite the degradation of RF induced by irradiation, the overall structure of the CS/RF film within the matrix remained unaffected.

The mechanical properties of films are crucial in determining their ability to preserve food integrity by standing handling and storage conditions. These properties are largely influenced by the distribution and density of intermolecular and intramolecular bonds within the chitosan matrix. In this study, both CS and CS/RF films were formulated with glycerol as plasticizer. Thus, the mechanical properties presented in Table 3 should be interpreted in the context of plasticizer incorporation. Generally, the addition of glycerol weakens tensile strength by disrupting chitosan’s polymeric interactions while enhancing flexibility, as evidenced by an increase in elongation at break, a trend consistent with previous findings [52]. No significant differences (*p* < 0.05) were observed in the mechanical properties between the CS and CS/RF film or between films subjected to irradiation (the stress–strain curves are provided in Figure S5). These results suggest that the inclusion of RF into the chitosan matrix did not induce significant structural changes, such as cross-linking or matrix reinforcement, which aligns with the ATR-FTIR analysis.

The mechanical stability of the CS/RF films under irradiation conditions reinforces their potential suitability for food packaging applications, as they maintain essential structural integrity while incorporating functional properties such as photo-responsiveness and antimicrobial activity.

### 2.3. Singlet Oxygen Production During Visible Light Exposure

**Figure 7:**
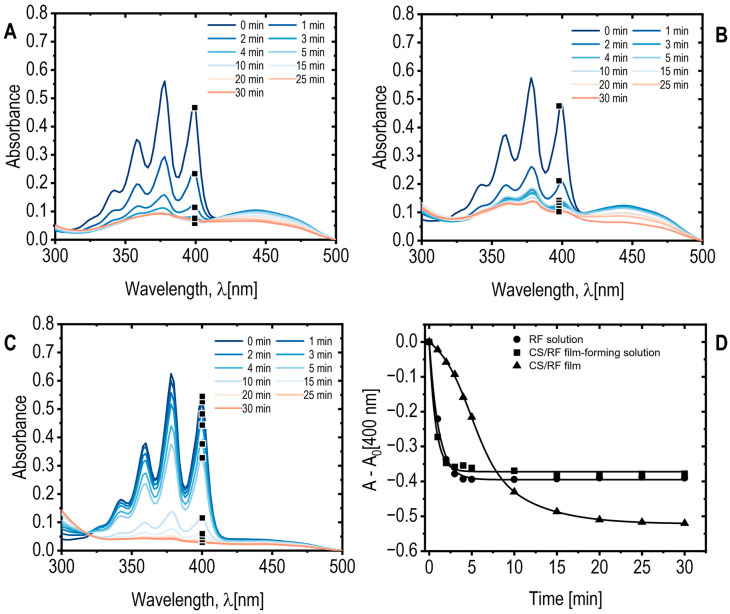
(**A**) ADMA absorption spectra for RF acetic acid solution; (**B**) CS/RF film-forming solution, and CS/RF film; (**C**) exposed to blue LED light for various durations. Note that only a selected wavelength range and specific exposure times are displayed for clarity; (**D**) ADMA absorbance at 400 nm, subtracting the absorbance at 0 min (A_0_), as a function of blue LED light exposure time. Selected data points are marked on the corresponding absorption spectra.

Reactive oxygen species (ROS) are recognized for their capacity to damage cell membranes via lipid peroxidation, serving as a pivotal function in photodynamic bacterial inactivation [53]. Consequently, the formation of ROS is highly relevant for antimicrobial applications. In this study, riboflavin (RF) was selected as the photosensitizer due to its excellent biocompatibility, affordability, and efficiency in generating ROS. Upon light absorption, RF undergoes excitation to the singlet state (S_1_) and subsequently transitions to the triplet state (T_1_) through intersystem crossing. The triplet state can promotes ROS formation via two distinct photophysical pathways: Type I, involving electron or hydrogen transfer reactions that yield radical species, and Type II, which leads to the formation of ^1^O_2_ [54]. The formation of ^1^O_2_ was evaluated for both CS/RF film-forming solution and the solid CS/RF films under blue LED light exposure in the presence of a chemical probe. The results were compared with those obtained from an RF acetic acid solution at an equivalent concentration (60 mg L^−1^). The water-soluble probe 9,10-anthracenediyl-bis(methylene)dimalonic acid (ADMA, 100 μM) was employed to quantify ^1^O_2_ generation, as it reacts irreversibly with ^1^O_2_ to form a non-absorbing endoperoxide. ADMA was selected because its absorption spectrum maximum below 400 nm prevents spectral overlap with the characteristic RF absorption band at 444 nm (Figure 7).

The kinetics and extent of ^1^O_2_ generation were determined by monitoring the decrease in ADMA absorbance (Figure 7A–D). Both the CS/RF film-forming solution and the RF solution exhibited similar reductions of approximately 40% decrease observed after 3 and 4 min of light exposition, respectively (Figure 7A,B,D). In contrast, the solid CS/RF film demonstrated a slower but more sustained decrease in ADMA absorbance, reaching approximately 50% after 20 min of irradiation (Figure 7C,D). Concurrent decreases in the RF absorption band at 444 nm indicated RF photodegradation alongside ADMA oxidation (Figure S6). No significant changes in ADMA absorbance were observed for the control sample exposed to blue LED light in the absence of RF or CS/RF, confirming that the observed decreases resulted exclusively from photoinduced ^1^O_2_ generation by the photosensitizer. Notably, in the solid CS/RF film, RF degradation continued even after ADMA oxidation had plateaued (Figure S6a,b), whereas both processes occurred simultaneously in the CS/RF films, reaching a steady state after approximately 20 min of irradiation. This behavior indicates enhanced and more stable ROS generation when RF is immobilized within the chitosan matrix, likely promoting efficient ^1^O_2_ production while minimizing RF release into the surrounding medium.

Although ROS quantification was conducted using both film-forming solutions and solid CS/RF films immersed in aqueous ADMA solutions for analytical purposes, the results demonstrate that ^1^O_2_ can be generated directly from the CS/RF films under light irradiation. In practical applications, these films function as solid-state photoactive materials, generating singlet oxygen at the film–air or film–moisture interface. This mechanism enables light-activated antibacterial effects on surfaces without requiring bulk aqueous environments. These findings further indicate the potential application of CS/RF films as light-activated antimicrobial materials, particularly in food packaging and surface protection.

### 2.4. Antimicrobial Activity of CS/RF Films: In Vitro Evaluation

The developed chitosan/riboflavin (CS/RF) films are primarily intended for application in perishable, protein-rich food products, such as meat, poultry, and fish, where *Pseudomonas* species are recognized as major spoilage microorganisms [55]. For this reason, *P. fluorescens*, a well-known Gram-negative bacterium frequently associated with the deterioration of refrigerated foods, was selected as a representative model organism. In this work, antibacterial activity was evaluated using a CFU-based contact assay rather than the conventional agar diffusion method, since the latter is not representative of how active food packaging materials function.

**Figure 8:**
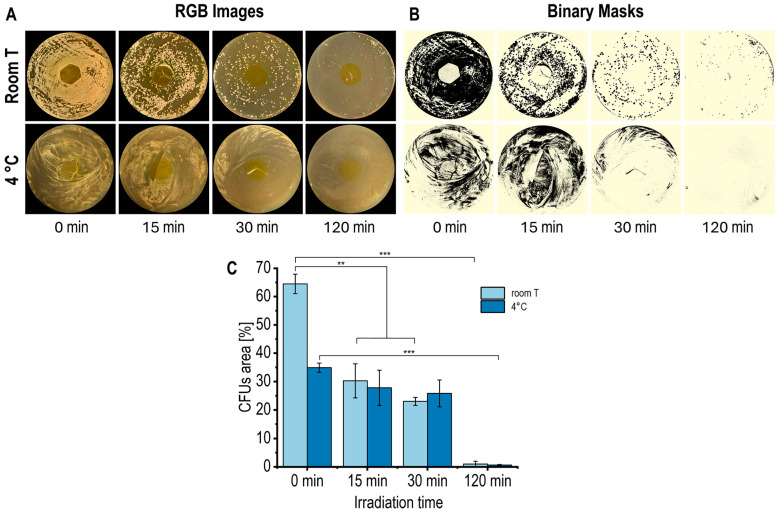
(**A**) RGB images, (**B**) corresponding binary masks, and (**C**) quantification of CFU coverage area (%) in Petri dishes with CS/RF film incubated at room temperature (for 48 h) and at 4 °C (for 120 h) and exposed to blue LED light for varying durations. Data in the bar chart are presented as mean ± standard deviation (error bars) of *n* = 3 (** *p* < 0.01, *** *p* < 0.001).

The antimicrobial activity of the CS/RF films was evaluated against *P. fluorescens* under different blue LED exposure times and incubation conditions (room temperature and 4 °C) to simulate typical food storage environments. After incubation period, each Petri dish was photographed to quantify the bacterial growth, which was expressed as the colony-forming unit (CFU) coverage area (%). The acquired RGB images were converted into the HSB color space, isolating the Brightness (B) channel for analysis. These images were then segmented into two distinct regions (ROIs): the plate surface and bacterial CFUs. A binarization was subsequently applied to quantify the percentage of the plate area covered by bacteria colonies. Figure 8A,B illustrate the image processing workflow, while Figure 8C shows the resulting CFU quantification.

The control Petri plates, with chitosan films without riboflavin (CS film), both unexposed (0 min) and exposed to blue LED light for 120 min, exhibited no notable antibacterial activity under the applied irradiation conditions (Figure S7). This outcome confirms that the intrinsic antibacterial effect of chitosan was minimal under the specific experimental conditions. In contrast, CS/RF films showed a gradual reduction in bacterial growth with extended exposure to blue LED light (Figure 8A–C), demonstrating that the photodynamically activated riboflavin was primarily accountable for the noted antibacterial action. After 120 min of irradiation (0.92 J cm^−2^), bacterial growth was reduced by approximately 97% at room temperature (after 48 h of incubation) and at 4 °C (after 120 h of incubation). Longer irradiation periods, corresponding to higher energy doses, further enhanced the antibacterial efficacy of the CS/RF films. These results are consistent with previous studies reporting that CS/RF composite films exhibit strong antibacterial activity against *Listeria monocytogenes*, *Vibrio parahaemolyticus*, and *Shewanella baltica* under extended photodynamic treatments up to 6.8 J cm^−2^ [15], which are approximately an order of magnitude more intense than those applied in the present work. Such comparisons highlight the importance of understanding the relationship between light energy dose, ROS production, and antimicrobial efficiency for optimizing the functional performance of CS/RF materials.

The photodynamic mechanism of the CS/RF films relies on the generation of photoexcited riboflavin molecules that can transfer energy to dissolved oxygen within the surrounding agar moisture, generating a gradient of ROS in the gas phase that spreads laterally around the film and acts through mechanisms not limited to simple diffusion within the agar matrix [20,56]. As a result, bacterial inhibition occurs across a wider area, but without forming a sharply defined halo (Figure 8). Similar approaches have been reported for other photodynamic and contact-active materials [57,58,59]. The significant reduction in viable colonies observed in Figure 8 confirms that the CS/RF films exhibit effective surface-confined photodynamic antibacterial activity under application-relevant conditions where microbial growth develops on food surfaces.

Overall, the antimicrobial activity demonstrated under these conditions supports the potential of CS/RF films for use in active packaging systems targeting refrigerated and fresh foods. Similar riboflavin-based photodynamic systems have shown promising antimicrobial performance in food preservation contexts [3,15]. Future research will aim to expand the microbial spectrum to include Gram-positive bacteria and fungi, thereby further validating the versatility and applicability of these films.

## 3. Materials and Methods

### 3.1. Reagents

Chitosan powder from crab shell with a molecular weight of 10–20 kDa and a degree of deacetylation ≥ 90% was obtained from Bio Basic Inc. (Markham, ON, Canada). Riboflavin (USP grade) and 9,10-Anthracenediyl-bis(methylene)dimalonic acid (ADMA) were purchased from Glentham Life Sciences (Corsham, UK). Sodium chloride (NaCl) and acetic acid (99.9%) were supplied by Sigma-Aldrich (Soborg, Denmark and St. Louis, MA, USA, respectively). Glycerol was sourced from Fluka (Buchs, Switzerland), and deuterium oxide (D_2_O) was acquired from Cambridge Isotope Laboratories, Inc. (Tewksbury, MA, USA). The psychrotropic *P. fluorescens LPF3* strain was previously isolated from raw milk and belongs to the bacterial collection of the Institute of Sciences of Food Production of the National Research Council of Italy (CNR-ISPA, Milan, Italy).

### 3.2. Preparation of Film-Forming Solutions and Films

Chitosan solution 1% (*w*/*v*) (CS) was prepared by dissolving chitosan in acetic acid 1% (*v*/*v*) under mechanical stirring overnight at room temperature. Glycerol, used as plasticizer, was then added at 25% (*w*/*w* of chitosan) and the mixture was mechanically stirred for 30 min. This mixture was used as film-forming solution to produce chitosan film, hereafter referred to as the CS film. For the CS/RF film, riboflavin (RF) was incorporated as a photosensitizer at a concentration of 60 mg/L, corresponding to its maximum solubility in the chitosan solution to ensure homogeneous dispersion and prevent precipitation of non-solubilized RF. The CS/RF solution was protected from light, stirred for an additional 2 h and then degassed using an ultrasonication bath for 30 min.

**Figure 9:**
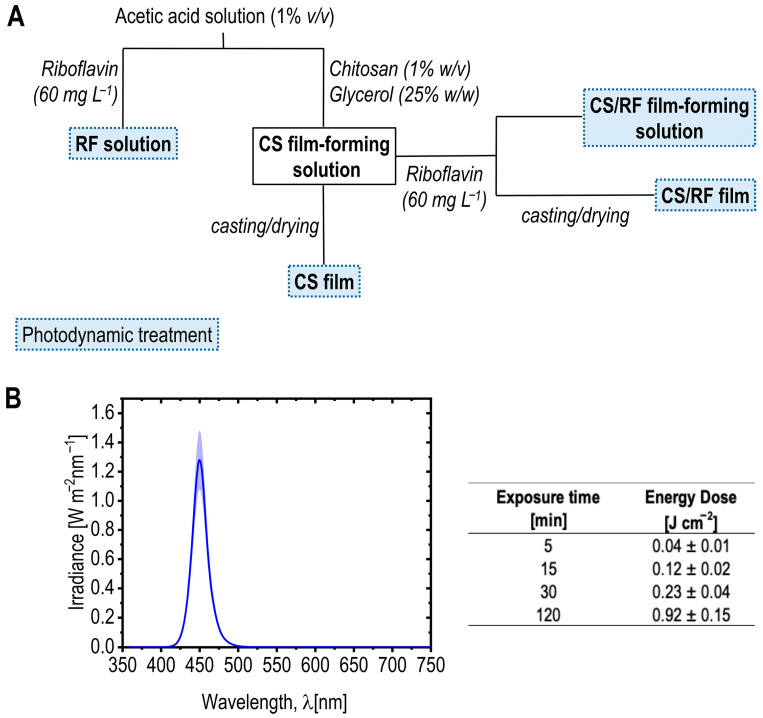
(**A**) Schematic representation of the experimental setup, illustrating sample preparation and the stages where photodynamic treatment (blue LED light, dashed boxes) was applied to investigate photodegradation kinetics. Samples names, as referred to throughout the paper, are highlighted in bold. (**B**) Spectral irradiance profile of blue LED light. The curve represents the mean irradiance from five measured points (dark blue line), with the standard deviation shown as the light blue shaded area. The table shows the total LED energy dose (E, J cm^−2^) calculated for selected exposure times using Equation (1).

Films CS and CS/RF were prepared by the solvent casting method with slight modifications [52]. The degassed film-forming solutions (i.e., CS and CS/RF) were poured into glass molds (10 cm × 10 cm × 0.3 cm) and dried under the hood at room temperature, protected from light, for 48 h. The resulting CS and CS/RF films were removed from the molds and stored in a light-protected desiccator at 23 °C and 50% relative humidity for at least 48 h according to ISO 187:2022 [60], to ensure moisture equilibration in the films before analyses. Figure 9A shows an overview of the experimental setup, highlighting sample names as referred to throughout the following sections, and the stages where the photodynamic treatment was applied to investigate the photochemical properties of RF in both solutions and films.

### 3.3. Photodynamic Treatment of Film-Forming Solutions and Films

A customized multispectral light-emitting diode (LED) system (Atena Lux srl, Italy) equipped with a wireless control interface (Casambi, Helsinki, Finland) consisting of 120 LEDs (Osram, Italy) was used, enabling precise adjustment of light intensity, wavelength, and exposure time for the photodynamic treatment of the RF acetic acid solution, the CS/RF film-forming solution, the dried CS/RF film, and their respective controls without RF. The samples were placed perpendicularly at 37 cm from the LED source. The spectral irradiance (W/m^2^ nm) was measured by a hand-held illuminance spectrophotometer (mod. CL-500A, Konica Minolta Sensing Europe, Cinisello Balsamo, Italy). The light source was operated on the blue channel, exhibiting an illuminance of 1261 lx at the sample position. The emission spectrum showed a main peak at 450 nm, and the chromaticity coordinates were x = 0.160 and y = 0.034, confirming the narrow-band blue character of the irradiation. The total LED energy dose applied during illumination was calculated according to Equation (1).

E = P t(1)

where E = Dose (energy density in J/cm^2^), P = irradiance (power density in W/cm^2^), and t = exposure time (in seconds).

Figure 9B presents the spectral irradiance profile of the blue LED light used in this study to investigate the kinetics of RF upon light activation in both film-forming solutions and films. The peak emission wavelength is the wavelength at which the LED emits its maximum light intensity, which in this case is 450 nm, falling within the absorption range of RF [26]. Various total LED energy doses (E, J cm^−2^) were applied during the characterization of the film-forming solution containing 60 mg L^−1^ of RF, as well as the dried films after casting (see the schematic representation of the experimental setup in Figure 9A). The table in Figure 9B provides the calculated energy doses at selected exposure times, referenced throughout the results and discussion section.

### 3.4. Study of Riboflavin Photodegradation in Film-Forming Solutions

#### 3.4.1. UV/Vis Spectral Absorbance

The photostability of the film-forming solutions was evaluated using a spectrophotometric method [36]. The solutions were placed in 100 mL flasks and exposed to LED light for varying durations, ranging from 5 to 240 min (i.e., corresponding to different total LED energy doses; Figure 9B). The photodegradation of RF (and potentially other compounds in the solutions) was assessed by recording the UV/Vis spectral absorbance. Spectral measurements were performed using a UV/Vis spectrophotometer (Lambda 25, Perkin Elmer, MA, USA), with absorbance recorded from 300 nm to 700 nm in 10 mm pathlength disposable cuvettes.

#### 3.4.2. NMR Measurements

The CS and CS/RF film-forming solutions before light exposure (0 min) and exposed to blue LED light (for 15, 30, and 120 min) were analyzed in duplicate using a high-field NMR spectrometer Bruker AV 600 (Bruker BioSpin GmbH, Ettinglen, Germany) operating at 600.33 MHz for ^1^H, equipped with a z-gradient probe. ^1^H NMR spectra were recorded at 25 °C. For each sample, 500 µL of the solution was mixed with 50 µL of D_2_O prior to analysis.

### 3.5. Determination of Singlet Oxygen Production in Film-Forming Solutions and Films

The production of singlet oxygen (^1^O_2_) upon exposure of the CS/RF film-forming solutions and films to photodynamic treatments was determined using 9,10-anthracenediyl-bis (methylene) dimalonic acid (ADMA) with some methodological modifications [15]. ADMA acted as a chemical probe for ^1^O_2_, reacting irreversibly with ^1^O_2_ to result in a decrease in ADMA absorption at a wavelength of 400 nm. The CS/RF film-forming solution, with a final RF concentration of 10 µM, was added to an ADMA solution (final concentration of 100 µM, dissolved in 0.85% physiological saline) and then exposed to LED light for varying durations (i.e., corresponding to different total LED energy doses). The decrease in ADMA absorption intensity was monitored using UV/Vis spectrophotometry, with samples placed in 10 mm pathlength disposable cuvettes. Control experiments were performed considering RF solutions added to ADMA solution and exposed to blue LED light. The production of singlet oxygen (^1^O_2_) of the CS/RF film upon exposure to photodynamic treatments was determined by adding a 3 mm × 3 mm CS/RF film into the ADMA solution (final concentrations 100 µM, dissolved in 0.85% physiological saline), and then exposed to blue LED light for varying durations up to 30 min. The ADMA absorption intensity at 400 nm was recorded. Control experiments were performed by exposing the ADMA solution to blue LED light in the absence of any sample.

### 3.6. Characterization of the Films

#### 3.6.1. Color

The color of CS/RF film was evaluated by recording the CIE L*, a*, and b* parameters using a portable colorimeter (mod. Chroma Meter CR-400, Konica Minolta, Sensing Europe, Cinisello Balsamo, Italy), illuminant D65 and observer 10°. The total color change (ΔE) and the yellowness index (YI) of CS/RF film exposed to blue LED light for varying durations up to 120 min (i.e., corresponding to different total LED energy doses) were calculated according to Equations (2) and (3) [61].

(2) $$∆E=\sqrt{\left[{\left(L^{*}-L_{0}^{*}\right)}^{2}+{\left(a^{*}-a_{0}^{*}\right)}^{2}+{\left(b^{*}-b_{0}^{*}\right)}^{2}\right]}$$

YI = (100 (C_x_ X − C_z_ Z))/Y (3)

where L*_0_, a*_0_ and b*_0_ are the color parameters of the CS/RF film before light exposure. The X, Y, and Z are the CIE tristimulus values, and C_x_ and C_z_ are illuminant- and observer-specific constants with values of 1.3013 and 1.1498 (for D65/10°), respectively. The mean values were determined by averaging measurements of 5 random points on the film.

#### 3.6.2. Transmittance Analysis

The ultraviolet and visible light barrier properties of the CS and CS/RF films before and after exposure to blue LED light were investigated by measuring their UV/Vis transmission using a high-performance UV/Vis spectrophotometer (mod. Lambda 650, Perkin Elmer, MA, USA). The spectra were scanned over the wavelength range of 200–800 nm. Film samples (3 cm × 3 cm) were fixed in the sample holder to ensure that the light beam passed uniformly over the film surface. The analyses were performed in triplicate.

The Opacity (O) was calculated by Equation (4) [42].

O = A_600_/L (4)

where A_600_ absorbance value at 600 nm and L is the average thickness (mm) of the films.

#### 3.6.3. Thickness

The thickness of the CS/RF films was measured using a high accuracy digital micrometer (Mitutoyo, Japan) with an accuracy of 0.001 mm. The mean thickness was determined by averaging measurements of 5 random points on the film.

#### 3.6.4. SEM Analysis

Scanning electron microscopy (SEM) images were acquired using a SEM-EDS JSM-IT500LA instrument (JEOL S.p.A., Basiglio, Italy). Prior to analysis, the samples were cut, mounted on aluminum stubs (Ø 12.5 mm; pin 3.2 × 8 mm) using carbon-based conductive double-sided adhesive tape, and subsequently gold-coated with a Scan Coat Six sputter coater (Edwards, 1996). Images were obtained in secondary electron (SE) mode under high vacuum (HV) conditions, with an accelerating voltage of 20 kV, an emission current of 2.54 μA, and a probe current (PC) of 40 μA.

#### 3.6.5. Thermal Properties

Thermogravimetric analysis (TGA) was conducted to assess the thermal stability of the CS and CS/RF films. Approximately 5 mg of each sample was analyzed using a TGA thermal analyzer (mod. Q500, TA Instruments, USA) at a heating rate of 10 °C/min, from room temperature (~30 °C) to 800 °C, under a nitrogen atmosphere with a flow rate of 50 cm^3^/min.

#### 3.6.6. Barrier Properties

The barrier properties of the CS and CS/RF films were assessed by measuring their oxygen and water vapor permeability. These measurements were performed on surface samples of 9 cm^2^ using an isostatic permeability analyzer (mod. TotalPerm, ExtraSolution^®^Srl, Pieve Fosciana, Italy). Data acquisition and analysis were conducted using the XS-Pro software (ExtraSolution^®^Srl, Pieve Fosciana, Italy). The instrument was equipped with a dual-sensor system: an electrochemical sensor for oxygen and an infrared sensor for water vapor. For the oxygen measurements, during the analysis, the instrument maintained a constant partial pressure difference of 1 bar between the two chambers of the permeation cell, using a nitrogen/hydrogen (99/1%) carrier gas flow, optimized for the material’s barrier characteristics. For the water vapor transmission rate, the driving force was determined by the temperature conditions and the relative humidity difference between the two cells. The oxygen transmission rate (O_2_TR, in cm^3^ m^−2^ day^−1^) was measured at 23 °C and 50% relative humidity, following the ASTM D3985 standard method (ASTM International, 2017). The water vapor transmission rate (WVTR, in g m^−2^ day^−1^) was determined at 23 °C and 65% relative humidity, according to the ASTM F1249-20 standard method (ASTM International, 2020). Permeability coefficients (KP, in cm^3^ µm m^−2^ day^−1^ bar^−1^ for gas or g µm m^−2^ day^−1^ bar^−1^ for water vapor) were obtained from O_2_TR and WVTR data using Equation (5).

KP_i_ = P_i_ · L = TR_i_/∆p (5)

where KP_i_ represents the permeability of the analyte (i = O_2_ or water vapor), P_i_ is the analyte permeance (defined as the ratio of the analyte transmission rate, TR_i_, to the difference in the partial pressure of the gas or vapor between the two sides of the film, Δp), and L is the film thickness.

#### 3.6.7. Attenuated Total Reflectance Fourier Transform Infrared Spectroscopy (ATR-FTIR)

ATR-FTIR spectroscopy analyses were performed on CS and CS/RF films using an FT-IR spectrometer (mod. Nicolet iS50, Thermo Scientific, WI, USA), employing an Attenuated Total Reflectance (ATR) accessory mode and a spherical diamond crystal, fixed at an incident angle of 45°. All spectra were collected in transmittance mode with a spectral resolution of 4 cm^−1^ and 64 scans recorded over the wavenumber range of 4000–600 cm^−1^. Each spectrum reported is the average of at least three spectra measured in different areas of the film. The ATR-IR spectra were normalized using Min-Max Normalization (0 to 1) to ensure consistency and enable meaningful comparisons, despite varying absorbance intensities.

#### 3.6.8. Mechanical Properties

The mechanical properties of CS and CS/RF films were evaluated using a tensile test conducted on a Universal Testing Machine (mod. Z005, ZwickRoell GmbH&Co, Ulm, Germany) equipped with a 5 kN load cell. The initial grip separation was set to 25 mm, and the crosshead speed was maintained at 150 mm min^−1^. Film strips with dimensions of 30 mm in length and 25 mm in width were used for the test. Stress–strain curves were generated and analyzed for the calculation of mechanical properties reported in Table 3. The stress, expressed in MPa, was calculated by dividing the maximum load (N) by the initial cross-sectional area (mm^2^) of the specimen.

### 3.7. In Vitro Antibacterial Activity and Image Analysis

The antibacterial activity of the CS/RF films under blue LED light exposure was evaluated against *Pseudomonas fluorescens* LPF3 using an image-based plate counting method adapted from the ISO 22196 standard [62]. The film was cut into 2 cm diameter disks and placed on Petri dishes containing Brain Heart Infusion agar (Biolife Italiana, Milan, Italy) seeded with bacterial suspension (10^4^–10^5^ CFUs per plate). Different plates were prepared and exposed to blue LED light for various durations (0, 15, 30, and 120 min). The plates were then incubated at room temperature for 48 h, and at 4 °C for 120 h, after which bacterial growth was quantified by counting colony-forming units (CFUs) using a developed image analysis method [63]. Control experiments were performed with Petri dishes containing the same load of bacterial colonies and exposed to blue LED light for 120 min. Each analysis was performed in duplicate.

Digital images of the plates were captured using a full-frame mirrorless digital camera (mod. Z6 II, Nikon, Tokyo, Japan) equipped with a 105 mm lens (mod. Nikkor 105), positioned vertically above the samples and connected to a PC for remote shooting. Pre-processing and processing of the RGB images, including thresholding, segmentation, and CFU coverage area quantification, was performed using ImageJ analysis software (version 1.48q, National Institutes of Health, Bethesda, MD, USA). For each Petri dish, the percentage of surface area covered by bacterial colonies was calculated.

### 3.8. Statistical Analysis

Significant differences among the results were evaluated by parametric analysis of variance (ANOVA) and Tukey multiple comparison, with a significance level of 95% (*p* < 0.05). When the Shapiro–Wilk test for normality and Levene’s test for homoscedasticity of the data yielded statistically significant results (*p* < 0.05), the Kruskal–Wallis non-parametric multiple range test and Holm’s stepwise adjustment with a significance level of 95% (*p* < 0.05) were used. Results are expressed by means ± standard deviations of replications. Statistical analysis was performed in R statistical software (R Foundation for Statistical Computing, Vienna, Austria).

## 4. Conclusions

This study evaluated the potential of chitosan-based (CS) films incorporated with riboflavin (RF) as a photo-active food packaging material with enhanced antimicrobial activity. The CS/RF films demonstrated promising properties for food preservation, with the ability to produce reactive oxygen species (ROS) under visible light exposure, particularly singlet oxygen (^1^O_2_), playing a significant role in microbial inactivation. Our findings suggest that the addition of RF into the chitosan matrix does not significantly alter the thermal stability and mechanical resistance of the film, making it suitable for practical food packaging applications. Moreover, CS/RF films maintained low oxygen permeability, reinforcing its potential for oxygen sensitive food preservation. Finally, the CS/RF films exhibited significant antimicrobial efficacy against a common food spoilage bacteria, with a reduction in bacterial growth by approximately 97% after 120 min of irradiation with possible positive effect on preserving food and extending its shelf-life. This enhanced antimicrobial effect was likely due to the photodynamic generation of ROS, as confirmed by the results of the ^1^O_2_ generation assay. The combination of its antimicrobial properties, ability to generate ROS under visible light, and the possibility for real-time monitoring of light exposure through color changes makes it a promising candidate for improving food preservation and safety. However, further research is necessary to validate its efficacy in real storage conditions, including variations in lighting and environmental factors, as well as its long-term performance in food preservation applications.

Our ongoing research focused on understanding how LED light doses, similar to those encountered during food storage, influence ROS generation and microbial inactivation. In this study, we utilized high-energy blue LED light, particularly in the 450 nm wavelength region, corresponding to the absorption peak of riboflavin. This specific wavelength was selected to maximize the photodynamic activation of the CS/RF film. Based on energy dose calculations at 450 nm, we estimated that typical white LED light used in supermarkets (4000 K, spectral profile and energy calculations detailed in Figure S8) would deliver an energy dose of approximately 0.9 J cm^−2^ after 50 h of exposure. This corresponds to the same level of photodynamic activation achieved within just 2 h of blue LED light exposure, indicating an acceleration factor of 25 under our experimental conditions. This equivalence suggests that the CS/RF packaging material could actively preserve food on supermarket shelves under standard lightning conditions by generating ROS over extended storage times. However, while these findings underscore the potential of CS/RF films as light-sensitive packaging materials, further validation is necessary to confirm their efficacy under real storage conditions, including variations in lightning conditions and packaging scenarios.

## Figures and Tables

**Table 1:** CIE L*, a*, and b* parameters, total color change (ΔE) and yellowness index (YI) of CS/RF film exposed to blue LED light for varying durations. Results are expressed as mean ± standard deviation of repetitions (*n* = 10).

Sample	L*	a*	b*	ΔE	YI
CS/RF 0 min	91.18 ± 0.49 ^a^	−11.25 ± 0.14 ^a^	53.65 ± 1.80 ^a^	/	70.34 ± 2.02 ^a^
CS/RF 15 min	90.69 ± 0.11 ^a^	−10.24 ± 0.06 ^b^	48.57 ± 1.21 ^b^	5.21 ± 1.18 ^a^	65.69 ± 1.34 ^b^
CS/RF 30 min	90.49 ± 0.27 ^a^	−9.75 ± 0.19 ^b^	46.25 ± 1.38 ^b^	6.86 ± 0.78 ^a^	64.38 ± 0.80 ^b^
CS/RF 120 min	90.24 ± 0.45 ^a^	−9.72 ± 0.38 ^b^	46.45 ± 0.66 ^b^	7.43 ± 0.77 ^a^	63.93 ± 0.24 ^b^

Values within columns with the same letters are not significantly different (*p* < 0.05).

**Table 2:** Oxygen and water vapor transmission rates (TR), and oxygen and water vapor permeability coefficients (KP) for CS and CS/RF films. Results are expressed as mean ± standard deviation of repetitions (*n* = 10).

Sample	O_2_TR, 23 °C, 1 Bar [cm^3^ m^−2^ Day^−1^]	KPO_2_ [cm^3^ µm m^−2^ Day^−1^ Bar^−1^]	WVTR, 23 °C, 65%RH [g m^−2^ Day^−1^]	KPWV [g µm m^−2^ Day^−1^ Bar^−1^]
CS film	3.65 ± 0.10 ^a^	100.50 ± 3.10 ^a^	1058 ± 59 ^a^	1.58 × 10^6^ ± 2.59 × 10^3 a^
CS/RF film	2.37 ± 0.53 ^a^	71.06 ± 15.89 ^a^	1113 ± 46 ^a^	1.75 × 10^6^ ± 2.38 × 10^5 a^

Values within columns with the same letters are not significantly different (*p* < 0.05).

**Table 3:** Mechanical properties of CS and CS/RF films before (0 min) and after 120 min of blue LED exposure. Data are presented as mean ± standard deviation (*n* = 10).

Sample	Young’s Modulus [MPa]	Yield Point		Modulus of Resilience [MPa]	Force at Break [kN/m]	Deformation at Break [mm]	Work at Break [MPa]
		Yield Strength	Yield Strain				
CS	662.6 ± 56.3 ^a^	39.2 ± 4.5 ^a^	0.08 ± 0.01 ^a^	1.0 ± 0.2 ^a^	1.2 ± 0.1 ^a^	13.6 ± 4.5 ^a^	21.6 ± 8.8 ^a^
CS/RF 0 min	614.7 ± 76.3 ^a^	41.2 ± 3.1 ^a^	0.09 ± 0.02 ^a^	1.3 ± 0.2 ^a^	1.3 ± 0.3 ^a^	7.7 ± 2.3 ^a^	19.6 ± 9.7 ^a^
CS/RF 120 min	695.2 ± 75.0 ^a^	41.2 ± 4.4 ^a^	0.14 ± 0.11 ^a^	1.3 ± 0.4 ^a^	1.2 ± 0.2 ^a^	11.2 ± 6.0 ^a^	22.2 ± 16.3 ^a^

Values within columns with the same letters are not significantly different (*p* < 0.05).

## Data Availability

All data are available in the manuscript.

## Supplementary Materials

The following supporting information can be downloaded at: https://www.mdpi.com/article/10.3390/molecules30214166/s1, Figure S1: Kinetic profile of RF photodegradation (H11 at δ 7.88 ppm) and the formation of LC (H3 at δ 7.64 ppm). Peaks assignments correspond to Figure 2; Figure S2: (a) Repeating units of chitosan showing the N-acetyl glucosamine (GlcNAc) and glucosamine (GlcN), (b) ^1^H NMR spectrum of CS, and (c) CS/RF film-forming solutions in 1% (v/v) CH_3_COOH/D_2_O before irradiation, with normalized integrated ^1^H signal values; Figure S3: (a) Oxygen transmission rate (O_2_TR) and (b) water vapor transmission rate (WVTR) measurement curves of CS and CS/RF film; Figure S4: ATR-FTIR spectra and zoomed-in fingerprint region for CS powder, CS and CS/RF films (a) before irradiation (0 min), and (b) at different irradiation times (0, 15 or 120 min); Figure S5: Stress–strain curves of CS/RF film unexposed to blue LED light (0 min) and exposed for 120 min. Curves of the same color represent repeated measurements; Figure S6: (a) UV–Vis spectra of film-forming solutions and film samples during ROS determination. (b) Kinetic curves evidencing the disappearance of riboflavin (444 nm band) observed even in the absence of ADMA oxidation (400 nm band); Figure S7: RGB images of control Petri dishes, without Riboflavin, unexposed (0 min) and exposed to blue LED light for 120 min and incubated at room temperature for 48 h; Figure S8: Spectral irradiance profile of white LED light. The curve represents the mean irradiance from five measured points (black line), with the standard deviation shown as the shaded area. The table shows the total LED energy dose (E, J cm^−2^) calculated for selected exposure times using Equation (1); Table S1: Description of ATR-IR absorption bands of chitosan powder, CS and CS/RF films without irradiation.

## Abbreviations

The following abbreviations are used in this manuscript:

RF	Riboflavin
CS	Chitosan
CS/RF	Chitosan/Riboflavin
PDI	Photodynamic Inactivation
ROS	Reactive Oxygen Species
^1^O_2_	Singlet Oxygen
S_0_	ground state
S_1_	singlet state
T_1_	triplet state
LED	Light-Emitting Diode
ADMA	9,10-anthracenediyl-bis (methylene)dimalonic acid
CDRF	cyclodehydroriboflavin
LC	lumichrome
LF	lumiflavin
FMF	formylmethylflavin
UV/Vis	Ultraviolet–Visible spectroscopy
^1^H NMR	Proton Nuclear Magnetic Resonance spectroscopy
SEM	Scanning Electron Microscopy
TGA	Thermogravimetric Analysis
ATR-FTIR	Attenuated Total Reflectance-Fourier Transform Infrared spectroscopy
O_2_TR	Oxygen Transmission Rate
WVTR	Water Vapor Transmission Rate
KP	Permeability coefficient
∆E	Total color change
YI	Yellowness Index
O	Opacity
RGB	Red–Green–Blue color space
CFUs	Colony-forming Units
