# Supplementary material for: Development of Photo-Active Chitosan-Based Films with Riboflavin for Enhanced Antimicrobial Food Packaging Applications
Source: Molecules. 2025 Oct 23;30(21):4166. doi: 10.3390/molecules30214166 (PMC12608488; doi:10.3390/molecules30214166)
Supplement: Supplementary file 1 [file molecules-30-04166-s001.zip › molecules-3912843-supplementary.pdf]

SUPPLEMENTARY MATERIALS

Development of Photo-Active Chitosan-Based Films with Riboflavin for Enhanced Antimicrobial Food Packaging Applications

Jessica Genovese [1,†], Daniele Maria Martins [1,†], Tiziana Silvetti [2], Milena Brasca [2], Daniela Fracassetti [1], Gigliola Borgonovo [1], Stefania Mazzini [1] and Sara Limbo [1,*]

[1] Department of Food, Environmental and Nutritional Sciences (DeFENS), Università degli Studi di Milano, Via G. Celoria 2, 20133 Milan, Italy; jessicagenovese1988@gmail.com (J.G.); daniele.martins@unimi.it (D.M.M.); daniela.fracassetti@unimi.it (D.F.); gigliola.borgonovo@unimi.it (G.B.); stefania.mazzini@unimi.it (S.M.)

[2] Institute of Sciences of Food Production, National Research Council (CNR-ISPA), Via G. Celoria 2, 20133 Milan, Italy; Tiziana.Silvetti@cnr.it (T.S.); milena.brasca@cnr.it (M.B.)

* Correspondence: sara.limbo@unimi.it (S.L.)

† These authors contributed equally to this work.

- **NMR Analysis**

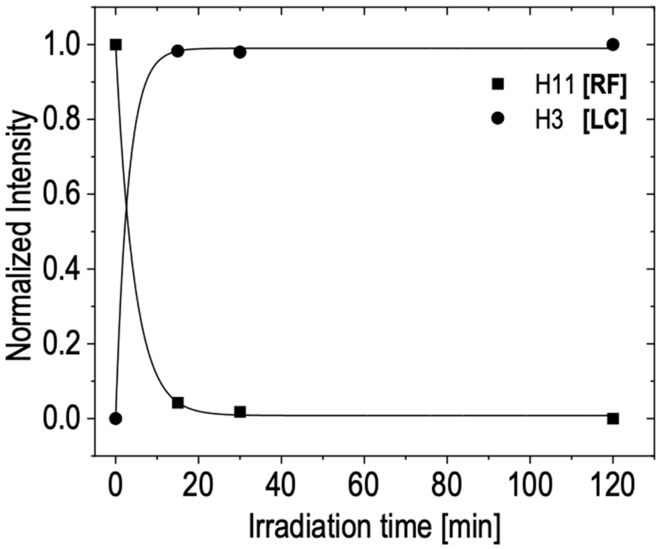

Figure S1. Kinetic profile of RF photodegradation (H11 at δ 7.88 ppm) and the formation of LC (H3 at δ 7.64 ppm). Peaks assignments correspond to Figure 2.

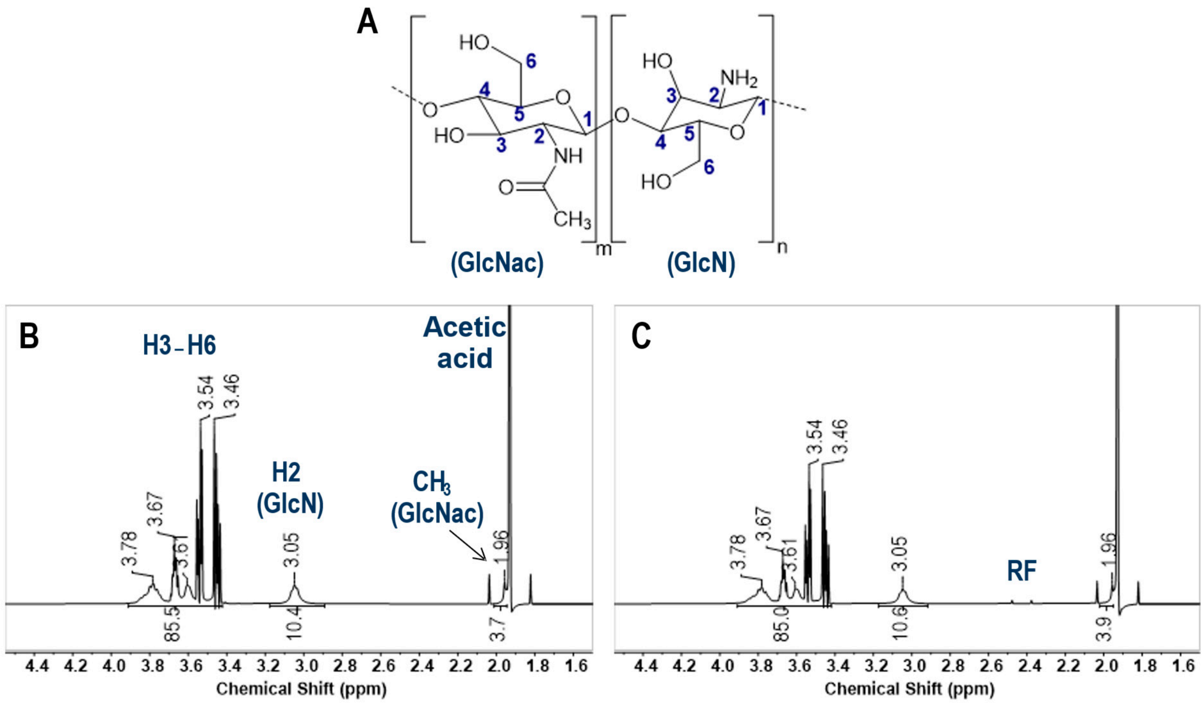

Figure S2. (**a**) Repeating units of chitosan showing the N-acetyl glucosamine (GlcNAc) and glucosamine (GlcN), (**b**) ^{1}H NMR spectrum of CS, and (**c**) CS/RF film-forming solutions in 1% (v/v) CH_3COOH/D_2O before irradiation, with normalized integrated ^{1}H signal values.

Figure S2 presents the spectra for both solutions, exhibiting characteristic signals of soluble chitosan corresponding to the N-acetyl glucosamine (GlcNac) and glucosamine (GlcN) units [39]. A peak at δ 1.96 ppm is attributed to the methyl protons of GlcNac, while the H2 protons of GlcN appears at δ 3.05 ppm (Figure S2 b,c). The region between δ 3.4-3.9 ppm contains overlapping resonances from the H3–H6 protons, along with the anomeric H1 protons of GlcN and GlcNAc. This region may also include overlapping signals from glycerol, which is present in the film-forming solution. The solvent peak (D_2O) is observed at approx. 4.7 ppm, while the acetic acid signal detected at δ 1.93 ppm (Figure S2) [38].

- **Barrier properties**

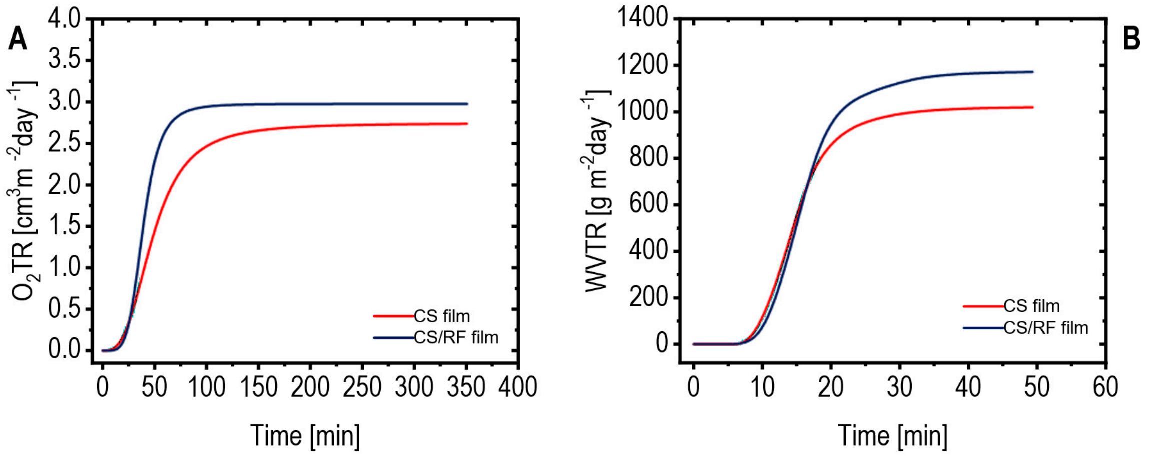

Figure S3. (**a**) Oxygen transmission rate (O_2TR) and (**b**)water vapor transmission rate (WVTR) measurement curves of CS and CS/RF film.

- **ATR-FTIR**

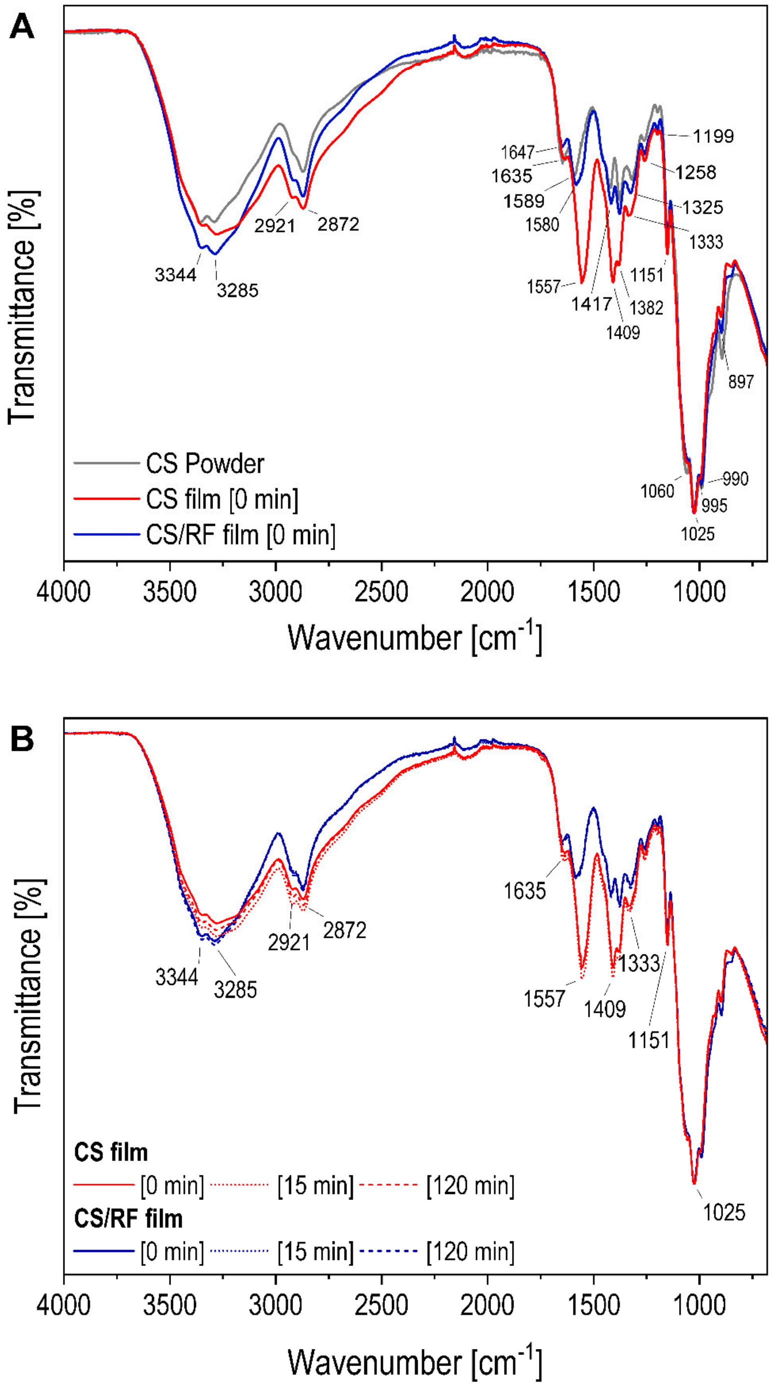

Figure S4. ATR-FTIR spectra and zoomed-in fingerprint region for CS powder, CS and CS/RF films (**a**) before irradiation (0 min); and (**b**) at different irradiation times (0, 15 or 120 min).

Table S1. Description of ATR-IR absorption bands of chitosan powder, CS and CS/RF films without irradiation.

Sample ID			Vibration mode assignment
**CS powder 10kDa**	**CS 0 min**	**CS/RF 0 min**	
**3356**	**3343**	**3344**	Stretching vibration of OH associated in pyranose ring overlapped with that of NH_2 associated in primary amines
**3292**	**3281**	**3285**	
	**3191**		
**2921**	**2921**	**2921**	Asymmetric stretching vibration of CH_2 in CH_2OH
**2872**	**2872**	**2872**	Asymmetric stretching vibration of CH in pyranose ring
**1646**	**1635**	**1647**	Stretching vibration of C=O in $NHCOCH_3$ (Amide I band)
**1589**	**1557**	**1580**	Bending vibration of NH_2 group and stretching vibration of C-N in $NHCOCH_3$ (Amide II band)
**1455**		**1455**	Bending vibration of CH_2 and C-H bond of the CH_3
**1419**	**1409**	**1417**	Bending vibration of CH_2 group in CH_2OH
**1375**	**1382**	**1377**	in-plane scissoring of CH_3 in $NHCOCH_3$
**1319**	**1333**	**1325**	Symmetric stretching vibration of -CH_3 in third amide and wagging of -CH_2 + OH deformation in plane
**1258**	**1258**	**1258**	Complex vibrations of NHCO (Amide III band)
**1197**	**1199**	**1199**	Stretching vibration of C-O bonds
**1150**	**1151**	**1151**	Symmetric stretching vibration of C-O-C
**1060**	**1060**	**1060**	Asymmetric stretching vibration of C-O-C (glycosidic linkage)
**1025**	**1025**	**1025**	Stretching vibration of C-O in secondary OH
**988**	**990**	**995**	Stretching vibration of C-O in primary OH

- Mechanical properties

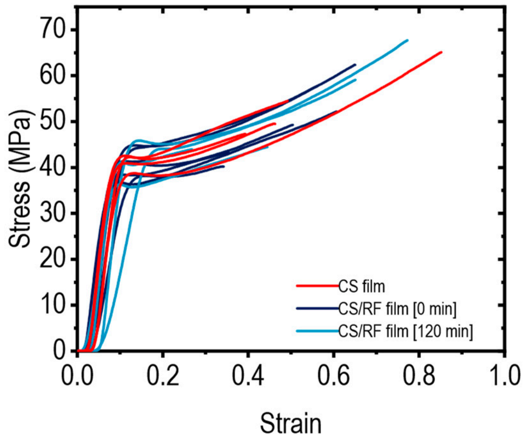

Figure S5. Stress-strain curves of CS/RF film unexposed to blue LED light (0 min) and exposed for 120 minutes. Curves of the same color represent repeated measurements.

- Reactive Oxygen Species

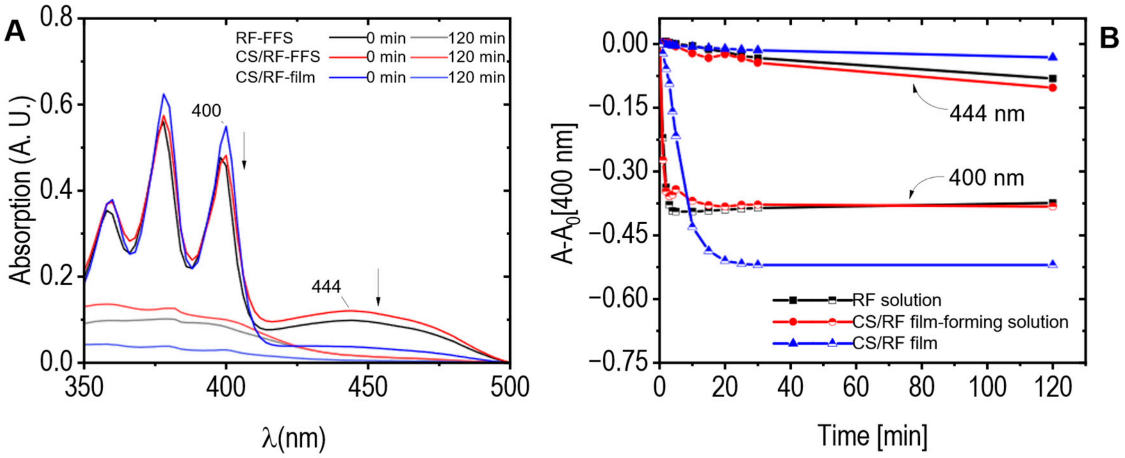

Figure S6 (**a**) UV-Vis spectra of film-forming solutions and film samples during ROS determination. (**b**) kinetic curves evidencing the disappearance of riboflavin (444 nm band) observed even in the absence of ADMA oxidation (400 nm band).

- Antimicrobial activity

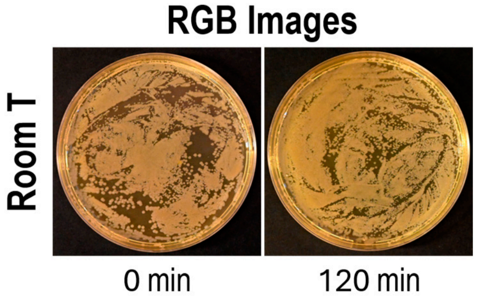

Figure S7. RGB images of control Petri dishes, without Riboflavin, unexposed (0 min) and exposed to blue LED light for 120 minutes and incubated at room temperature for 48 h.

- **Spectral irradiance profile of commercial white LED light**

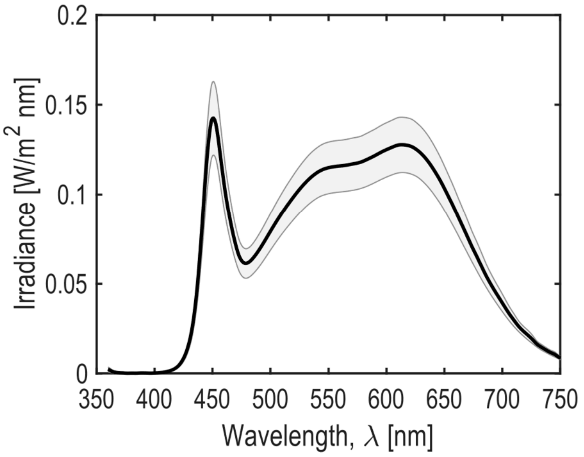

Exposure time [min]	Energy Dose [J cm^{-2}]
5	0.002
30	0.01
120	0.04
3000	0.95

Figure S8. Spectral irradiance profile of white LED light. The curve represents the mean irradiance from five measured points (black line), with the standard deviation shown as the shaded area. The table shows the total LED energy dose (E, J cm^{-2}) calculated for selected exposure times using Equation 1.
